# Neurofilament light chain: a biomarker for genetic frontotemporal dementia

**DOI:** 10.1002/acn3.325

**Published:** 2016-07-01

**Authors:** Lieke H. Meeter, Elise G. Dopper, Lize C. Jiskoot, Raquel Sanchez‐Valle, Caroline Graff, Luisa Benussi, Roberta Ghidoni, Yolande A. Pijnenburg, Barbara Borroni, Daniela Galimberti, Robert Jr Laforce, Mario Masellis, Rik Vandenberghe, Isabelle Le Ber, Markus Otto, Rick van Minkelen, Janne M. Papma, Serge A. Rombouts, Mircea Balasa, Linn Öijerstedt, Vesna Jelic, Katrina M. Dick, David M. Cash, Sophie R. Harding, M. Jorge Cardoso, Sebastien Ourselin, Martin N. Rossor, Alessandro Padovani, Elio Scarpini, Chiara Fenoglio, Maria C. Tartaglia, Foudil Lamari, Christian Barro, Jens Kuhle, Jonathan D. Rohrer, Charlotte E. Teunissen, John C. van Swieten

**Affiliations:** ^1^ Alzheimer Center Rotterdam and Department of Neurology Erasmus Medical Center PO Box 2040, 3000 CA Rotterdam The Netherlands; ^2^ Alzheimer's Disease and Other Cognitive Disorders Unit Department of Neurology Hospital Clínic Institut d'Investigació Biomèdica August Pi i Sunyer Villarroel, 170 Barcelona 08036 Spain; ^3^ Division of Neurogeriatrics Department NVS, Karolinska Institutet Center for Alzheimer Research Huddinge 141 57 Sweden; ^4^ Department of Geriatric Medicine Karolinska University Hospital‐ Huddinge Stockholm 141 86 Sweden; ^5^ Molecular Markers Laboratory IRCCS Centro San Giovanni di Dio Fatebenefratelli via Pilastroni 4 Brescia 25125 Italy; ^6^ Alzheimer Center and Department of Neurology Neuroscience Campus Amsterdam VU University Medical Center PO Box 7057, 1007 MB Amsterdam The Netherlands; ^7^ Neurology Unit Department of Clinical and Experimental Sciences Centre for Neurodegenerative Diseases University of Brescia Brescia Italy; ^8^ University of Milan Fondazione Ca’ Granda IRCSS Ospedale Policlinico Milan Italy; ^9^ Département des Sciences Neurologiques Clinique Interdisciplinaire de Mémoire (CIME) CHU de Québec Université Laval Québec Canada; ^10^ Division of Neurology Department of Medicine Sunnybrook Health Sciences Centre University of Toronto Toronto Canada; ^11^ Hurvitz Brain Sciences Research Program Sunnybrook Research Institute Toronto Canada; ^12^ Neurology University Hospitals Leuven Herestraat 49 Leuven Belgium; ^13^ Laboratory for Cognitive Neurology Department of Neurosciences KU Leuven Leuven Belgium; ^14^ Institut du Cerveau et de la Moelle épinière (ICM) Inserm U1127 CNRS UMR 7225 Sorbonne Universités Université Pierre et Marie Curie Univ Paris 06 UPMC‐P6 UMR S 1127 ‐ Hôpital Pitié‐Salpêtrière Paris France; ^15^ Centre de Référence des Démences Rares AP‐HP Hôpital de la Pitié‐Salpêtrière Paris France; ^16^ Département des maladies du système nerveux AP‐HP Hôpital de la Pitié‐Salpêtrière Paris France; ^17^ Department of Neurology Ulm University Ulm Germany; ^18^ German FTLD consortium Department of Neurology University of Ulm Ulm Germany; ^19^ Department of Clinical Genetics Erasmus Medical Center PO Box 2040, 3000 CA Rotterdam The Netherlands; ^20^ Institute of Psychology Leiden University Leiden The Netherlands; ^21^ Department of Radiology Leiden University Medical Center Leiden The Netherlands; ^22^ Division of clinical geriatrics Deptartment NVS Karolinska Institutet Center for Alzheimer Research Huddinge 141 57 Sweden; ^23^ Dementia Research Centre Department of Neurodegenerative Disesase Institute of Neurology University College London WC1N 3BG London United Kingdom; ^24^ Translational Imaging Group Centre for Medical Image Computing University College London NW1 2HE London United Kingdom; ^25^ Laboratoire de Biochimie AP‐HP Hopital Pitié‐Salpétrière Paris France; ^26^ Tanz Center for Research in Neurodegenerative Diseases University of Toronoto Toronoto Canada; ^27^ Neurology Departments of Medicine Biomedicine and Clinical Research University Hospital Basel Basel Switzerland; ^28^ Neurochemistry Lab and Biobank Department of Clinical Chemistry Neuroscience Campus VU University Medical Center PO Box 7057, 1007 MB Amsterdam The Netherlands; ^29^ Department of Clinical Genetics VU University Medical Center PO Box 7057, 1007 MB Amsterdam The Netherlands

## Abstract

**Objective:**

To evaluate cerebrospinal fluid (CSF) and serum neurofilament light chain (NfL) levels in genetic frontotemporal dementia (FTD) as a potential biomarker in the presymptomatic stage and during the conversion into the symptomatic stage. Additionally, to correlate NfL levels to clinical and neuroimaging parameters.

**Methods:**

In this multicenter case–control study, we investigated CSF NfL in 174 subjects (48 controls, 40 presymptomatic carriers and 86 patients with *microtubule‐associated protein tau* (*MAPT*), *progranulin* (*GRN*), and *chromosome 9 open reading frame 72* (*C9orf72*) mutations), and serum NfL in 118 subjects (39 controls, 44 presymptomatic carriers, 35 patients). In 55 subjects both CSF and serum was determined. In two subjects CSF was available before and after symptom onset (converters). Additionally, NfL levels were correlated with clinical parameters, survival, and regional brain atrophy.

**Results:**

CSF NfL levels in patients (median 6762 pg/mL, interquartile range 3186–9309 pg/mL) were strongly elevated compared with presymptomatic carriers (804 pg/mL, 627–1173 pg/mL, *P* < 0.001), resulting in a good diagnostic performance to discriminate both groups. Serum NfL correlated highly with CSF NfL (*r*_*s*_= 0.87, *P* < 0.001) and was similarly elevated in patients. Longitudinal samples in the converters showed a three‐ to fourfold increase in CSF NfL after disease onset. Additionally, NfL levels in patients correlated with disease severity, brain atrophy, annualized brain atrophy rate and survival.

**Interpretation:**

NfL in both serum and CSF has the potential to serve as a biomarker for clinical disease onset and has a prognostic value in genetic FTD.

## Introduction

Mutations in the *microtubule‐associated protein tau* (*MAPT*), *progranulin* (*GRN*) or *chromosome 9 open reading frame 72* (*C9orf72*) genes are major causes of genetic frontotemporal dementia (FTD) and are associated with considerable clinical heterogeneity.^1^, ^2^, ^3^, ^4^, ^5^ The presymptomatic stage offers a unique window to study the earliest disease stages.^6^ Changes in neuroimaging biomarkers have been found in presymptomatic FTD, similar to findings in familial Alzheimer's disease (AD) and Huntington's disease.^6^, ^7^, ^8^, ^9^ However, fluid biomarkers determining disease onset and progression are lacking, which are essential for forthcoming trials on disease modifying treatments. Neurofilament light chain (NfL) in cerebrospinal fluid (CSF) is elevated in FTD, and other neurodegenerative diseases such as amyotrophic lateral sclerosis (ALS), AD, and vascular dementia, and dynamically decreases in response to anti‐inflammatory treatments in multiple sclerosis.^10^, ^11^, ^12^, ^13^, ^14^ In contrast, small series of presymptomatic carriers of FTD‐causing mutations have shown low CSF NfL levels.^10^, ^15^ NfL is one of the three subunits of neurofilaments, which are the major constituent of the neuroaxonal cytoskeleton and are essential for axonal growth, transport, and signalling pathways.^16^, ^17^ CSF NfL has been correlated with disease severity, disease progression, and brain atrophy in neurodegenerative diseases.^10^, ^13^, ^18^ Blood‐derived NfL levels have proven to highly correlate with CSF NfL in ALS.^18^ An important question is whether NfL levels may serve as a biomarker for conversion from presymptomatic to symptomatic genetic FTD and be useful in tracking disease severity and progression.

To evaluate the potential of NfL levels as a biomarker in genetic FTD, we determined CSF and serum NfL in presymptomatic carriers and patients with pathogenic mutations in *MAPT, GRN* or *C9orf72*, and correlated these levels with clinical and neuroimaging measures.

## Methods

### Subjects

For this study, three subject groups were included from 11 centers collaborating in the Genetic FTD Initiative (GENFI)^19^: (1) patients with FTD caused by a pathogenic mutation in *GRN*,* MAPT* or *C9orf72* (*n* = 102); (2) presymptomatic carriers of a pathogenic mutation (*n* = 63); and (3) cognitively healthy subjects without mutation (controls, *n* = 73). A pathogenic *C9orf72* expansion was defined as more than 30 repeats.^5^ For *GRN*, only nonsense mutations were included (Table 1), for *MAPT*, published pathogenic mutations and those predicted as pathogenic were taken into account (software package Alamut v2.6.1, Interactive Biosoftware, Rouen, France; Table 1). Participants were recruited as part of GENFI (*n* = 126) or ascertained before participation in GENFI (*n* = 112). Participants were either patients with a mutation, or known presymptomatic carriers, or 50% at‐risk individuals (presymptomatic carriers and controls), or cognitively healthy family members without a mutation (controls). At‐risk individuals are first‐degree relatives of a known carrier of a pathogenic mutation. Genotyping of all participants was performed at local sites and clinical investigators were blinded for the mutation status of at‐risk individuals. At‐risk individuals and control subjects underwent neuropsychological examination. Subjects were categorized as presymptomatic or symptomatic according to criteria at the time of inclusion.^20^, ^21^, ^22^ At‐risk individuals were followed yearly or two yearly to assess conversion into symptomatic FTD. We defined conversion as the presence of symptoms of behavioral variant FTD (bvFTD), primary progressive aphasia (PPA) or amnestic FTD as reported by informants and supported by neuropsychological assessment and neuroimaging. Disease onset in patients (*n* = 102) and converters (*n* = 4) was defined as the moment of first symptoms noted by a caregiver. In presymptomatic carriers, estimated time from onset was calculated as age at sample collection minus mean familial age at onset, resulting in a negative measure in carriers younger than the estimated onset derived from onset ages in their family.^6^ Mini‐Mental State Examination (MMSE) was used to measure global cognition,^23^ disease severity was assessed by the Clinical Dementia Rating scale (CDR) including, if available, the sum of boxes (CDR‐SB);^24^ we only considered scale measurements within 90 days of biosample collection. In seven subjects (five CSF, one serum, one both; five *C9orf72*, two *GRN* mutations)^25^ ALS‐symptoms were present at sample collection; five of them met El Escorial criteria at collection, the other two 6 months after collection.^26^ No ALS‐patients without FTD symptoms were included.

**Table 1:** Subject characteristics

	Controls	Presymptomatic carriers			Patients			*P*‐value
Number	71	62			101			
Male gender	29 (41%)	23 (37%)			49 (49%)			0.32
Age at collection, years (IQR)	54 (43–61)	49 (42–57)			59 (56–65)			<0.0001
Age at onset, years (range)	–	55 (46–70)^a^			56 (39–76)^b^			0.84
Disease duration, years (IQR)	–	–			2.0 (1.3–3.4)			
Time to onset or estimated onset, years (IQR)	–	7.3 (2.5 – 13.2)^a^			–			
MMSE (IQR)	29 (29–30)	30 (29–30)			25 (21–28)			<0.0001
Concomitant ALS	0	0			7			0.005
Gene‐specific information		*GRN*	*C9orf72*	*MAPT*	*GRN*	*C9orf72*	*MAPT*	
Number per gene		34^c^	14	14^d^	53^e^	29	19^f^	
Age at collection, years (IQR)		55 (48–58)	45 (42–49)	41 (36–49)	60 (57–65)	61 (55–68)	57 (53–59)	
Age at onset, years (range)					58 (47–76)	55 (39–75)	53 (42–70)	
Disease duration, years (IQR)					1.8 (1.1–2.6)	3.0 (2.0–5.0)	2.1 (1.5–3.7)	0.008
Time to onset or estimated onset, years (IQR)		5.8 (1.6–11.0)	11.5 (5.9–14.8)	7.3 (3.3–15.8)				0.19

Values are displayed as median (IQR). In the case of multiple samples in one subject, characteristics at first collection are displayed. IQR, interquartile range; ALS, amyotrophic lateral sclerosis; MMSE, mini‐mental state examination.

a Four presymptomatic subjects converted during follow‐up into symptomatic stage after collection (2 with CSF, 1 with serum and 1 with CSF and serum).

b In two patients the age at onset was unknown.

c 17 Ser82fs, 8 Gln125X, 5 Gly35fs, 2 Val411fs, 2 Cys416fs.

d 8 Pro301Leu, 3 Gly272Val, 1 Arg406Trp, 1 Leu135Arg, 1 Ser320Phe.

e 16 Thr272fs, 7 Ser82fs, 4 Gly35fs, 4 IVS1+5G>C, 3 Cys366fs, 3 Tyr294X, 2 Gln125X, 1 c.708+6+9delTGAG, 1 Gln257fs, 1 Val279fs, 1 Gln341X, 1 Thr278fs, 1 Cys314X, 1 c.709‐3C>G homozygous, 1 Gln130fs, 1 Cys149fs, 1 Cys157fs, 1 Cys315X, 1 Asn188fs, 1 Val200fs, 1 Pro127fs.

f 10 Pro301Leu, 2 Gly272Val, 3 Arg406Trp, 1 Leu315Arg, 1 Val337Met, 1 Val287Ile, 1 Ser305Thr.

Local ethics committees at each site approved the study and all participants (or a legal representative) provided written informed consent at enrollment.

### Procedures

CSF (*n* = 179) was collected according to standardized local procedures. Serum samples were collected from Dutch participants only (*n* = 120). Both CSF and serum collection within 1 year were available in 57 out of 61 subjects with both CSF and serum (same day *n* = 37, range 0–360 days). Longitudinal CSF samples were available in five subjects, including converters; in one converter a third CSF sample was available. T1‐weighted MRI‐images within 6 months of CSF collection were available in 101 subjects and a follow‐up scan in 22 subjects. Detailed data on available biosamples and MRI scans in the three subgroups after exclusion of outliers (see Statistical analyses) are presented in Figure S1. Gray matter volumes were determined by anatomical parcellation of the whole brain, using a multiatlas segmentation propagation approach,^27^, ^28^ with the anatomical definitions following the brainCOLOR protocol for the cortical regions and Neuromorphometrics protocol for subcortical regions and other structures.^29^, ^30^ Regions‐of‐interest were combined to calculate gray matter volumes of the frontal, temporal, parietal, occipital, cingulate, and insular cortices.^28^ Whole‐brain volumes were calculated by combining all regions from the automated brain segmentation method.^30^ All volumes are presented as percentage of total intracranial volume (TIV). Atrophy rates were calculated as the percentage decrease in volume per year relative to baseline.

### Laboratory methods

Measurements of NfL (in CSF and serum) were performed in one laboratory (of CET respectively JK), blinded to clinical information and mutation status. CSF NfL was measured in duplicates using the enzyme‐linked immunosorbent assay (ELISA) of Uman Diagnostics (Umeå, Sweden), according to the manufacturer's instructions over four different batches. Median intra‐assay coefficient of variation (CV) was 0.8% (range 0–66.5%), inter‐assay variability was below 20%. Serum NfL concentrations were measured in duplicate by an earlier described, slightly modified, electrochemiluminescence immunoassay with antibodies identical to those used in the CSF ELISA (Data S1).^31^, ^32^

### Statistical analyses

Statistical analyses were performed in SPSS 21.0 for Windows (Armonk, NY, USA) and GraphPad Prism 6 (La Jolla, California, USA) applying a significance level of *P* < 0.05. NfL values with an intra‐assay CV of >20% (*n* = 1) and outliers (values > three standard deviations from the mean: four CSF and two serum samples) were excluded. CSF and serum NfL were analyzed using nonparametric tests (Mann–Whitney *U* tests and Kruskal–Wallis with Dunn's post hoc tests). Since the data were not normally distributed, and log‐transformation did not normalize the data, square root transformed CSF and serum NfL were used to correct for age in all subjects and disease duration in patients using analysis of covariance (ANCOVA) with post hoc Bonferroni corrections where appropriate. Spearman's correlation coefficient (*r*_*s*_) was used to correlate serum with CSF NfL, NfL levels with clinical measures and CSF NfL with brain volumes, the latter also with correction for gender and study site (partial rank correlations). Diagnostic performance was assessed by areas under the curve (AUC) with 95% confidence intervals (CIs) obtained by receiver operating characteristic (ROC) analyses, with optimal cut‐off levels at the highest Youden's index (sensitivity + specificity‐1).^33^ In analogy to the study of Lu et al.,^18^ survival in patients was compared between NfL tertiles by Kaplan–Meier curves and Cox regressions adjusted for age and disease duration. NfL concentrations are described as medians.

## Results

### Demographic and clinical data

The total group of 234 subjects consisted of 101 patients (53 *GRN*, 29 *C9orf72*, 19 *MAPT*), 62 presymptomatic carriers (34 *GRN*, 14 *C9orf72*, 14 *MAPT*) and 71 controls (Table 1, Fig. S1). Patients were older than presymptomatic carriers (*P* < 0.001) and controls (*P* < 0.001). *GRN* and *C9orf72* patients were older than *MAPT* patients (*P* = 0.01 and *P* = 0.04 respectively). The age at onset in patients was highly variable ranging between 39 and 76 years and several presymptomatic carriers were past their estimated age at onset. However, 50% percent of the patients had an onset between 52 and 62 years; and the age of both converters was close to the estimated onset age. The disease duration in *C9orf72* patients was longer than in *GRN* patients (*P* = 0.007). The clinical presentation was bvFTD (*n* = 60), PPA (*n* = 17), FTD‐ALS (*n* = 7), predominant memory phenotype (*n* = 4), mild cognitive impairment (*n* = 4), progressive supranuclear palsy or corticobasal syndrome (*n* = 2), and dementia not otherwise specified (*n* = 7).

### NfL in CSF and in serum

**Figure 1:**
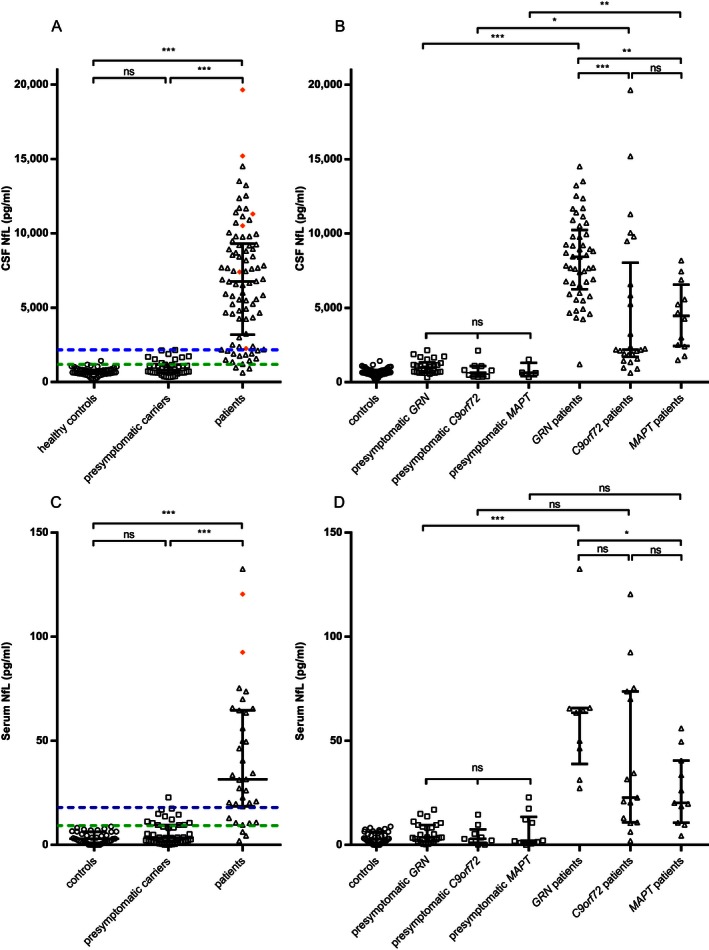
Neurofilament light chain (NfL) levels in presymptomatic carriers and patients. NfL in (A) cerebrospinal fluid (CSF) and (C) serum by controls, presymptomatic carriers and patients; patients with concomitant amyotrophic lateral sclerosis are displayed as filled orange diamonds. Upper blue dashed lines represent the cut‐off line to separate presymptomatic carriers from patients at 2165 pg/mL for CSF (sensitivity 84%, specificity 100%) and at 18.0 pg/mL for serum (sensitivity 77%, specificity 98%). Lower green dashed lines represent the cut‐off line to separate controls from patients at 1190 pg/mL for CSF (sensitivity 97%, specificity 98%) and at 9.3 pg/mL for serum (sensitivity 91%, specificity 100%). NfL levels in (B) CSF and (D) serum specified by genetic group and clinical stage. Significances from the analysis of covariance analyses are displayed (corrected for age in all comparisons and additionally for disease duration in the comparisons between affected genes in patients). In Figure S3, graphs of the transformed data are shown. Ns, not significant; *P ≤ 0.05; **P ≤ 0.01; ***P ≤ 0.001.

CSF NfL levels in patients (6762 pg/mL) were more than eight times higher than in presymptomatic carriers (804 pg/mL) and controls (650 pg/mL, both *P* < 0.001, Fig. 1A), without a difference between the latter two groups (*P* = 0.46, Fig. S2A). The elevation was confirmed after genetic stratification (Fig. 1B). *GRN* patients had higher CSF NfL levels than *C9orf72* and *MAPT* patients (*P* < 0.001 and *P* = 0.004 respectively, Fig. 1B). CSF NfL did not differ between the three presymptomatic groups (*P* = 0.17, Fig. S2B). Correction for age in all subjects and disease duration in patients on square root transformed CSF NfL yielded similar *P*‐values as without transformation, except for presymptomatic *C9orf72* cases versus *C9orf72* patient (before correction *P* < 0.001, after correction *P* = 0.04, all corrected *P*‐values are displayed in Figure 1 and transformed data is presented in Fig. S3).

NfL levels in serum showed a similar pattern as in CSF, with higher levels in patients (31.5 pg/mL) than in presymptomatic carriers (3.5 pg/mL, *P* < 0.001) and controls (2.9 pg/mL, *P* < 0.001, Fig. 1C), without a difference between the latter two groups (Fig. S2C). Consistently, the elevation was confirmed after genetic stratification. *GRN* patients had higher serum NfL levels than *MAPT* patients (*P* = 0.03, Fig. 1D), both did not differ from *C9orf72* patients. Serum NfL did not differ between the three presymptomatic groups (*P* = 0.76, Fig. S2D). Correction for age and disease duration showed similar results, except for the difference between presymptomatic carriers and patients which showed only a trend for the *MAPT* and *C9orf72* mutations (both *P* = 0.11, Fig. 1D and Fig. S3C and S3D), probably due to the small groups.

### Correlation between CSF and serum NfL

**Figure 2:**
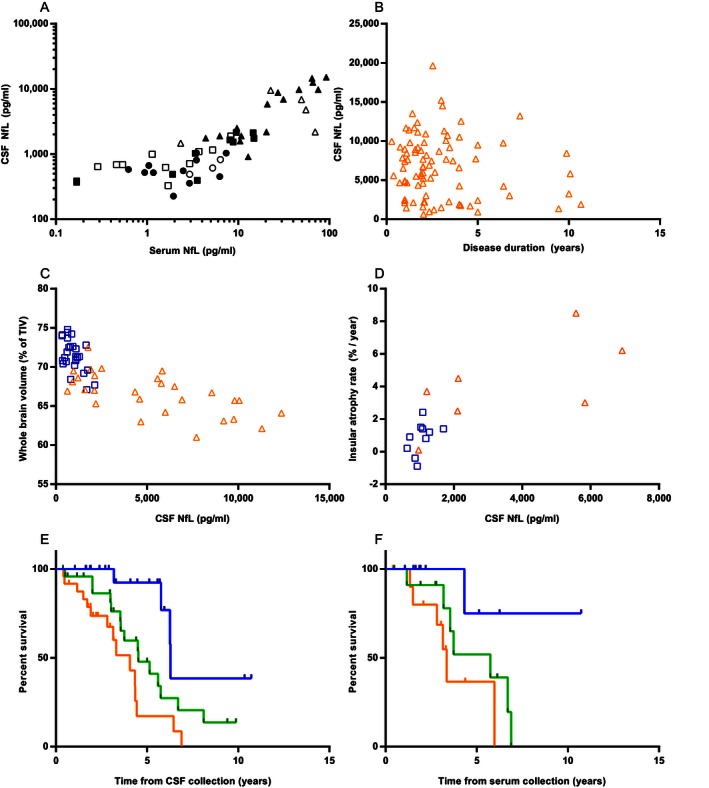
Correlations between neurofilament light chain (NfL) levels and clinical or imaging data. (A) Correlation between serum and cerebrospinal fluid (CSF) NfL, circles represent controls, squares represent presymptomatic carriers and triangles represent patients; filled data points are collected on the same day; a log‐scale is used for display purposes, one sample had a serum NfL of 0 pg/mL and is thus excluded from the graph, but not from the analysis. (B) Correlation of CSF NfL with disease duration in patients (orange triangles). Correlations between CSF NfL and (C) whole‐brain volume and (D) insular annualized atrophy rate in presymptomatic carriers (blue squares) and patients (orange triangles). Kaplan–Meier curves of (E) all patients with CSF available and (F) all patients with serum available; NfL levels were stratified into tertiles: the blue upper lines represent the lowest tertiles, the green middle lines the middle tertiles and the orange lower lines the highest tertiles; information on survival was available in 72 out of 86 patients with CSF and all patients with serum (*n* = 35).

CSF NfL correlated strongly with serum NfL (Fig. 2A, entire group *r*_*s*_= 0.87, *P* < 0.001). The correlations were strongest in carriers (patients *r*_*s*_ = 0.77, *P* < 0.001 and presymptomatic carriers *r*_*s*_ = 0.83, *P* < 0.001), whereas controls showed only a trend (*r*_*s*_ = 0.50, *P* = 0.06). Sample sets collected on the same day showed slightly, although not significantly, stronger correlations (*n* = 37, entire group *r*_*s*_= 0.90, presymptomatic carriers *r*_*s*_= 0.90, and patients *r*_*s*_= 0.86, all *P* < 0.001).

### Correlation with demographical and clinical characteristics

Age correlated with CSF NfL levels in presymptomatic carriers and controls (*r*_*s*_= 0.79, *P* < 0.001 respectively *r*_*s*_= 0.58, *P* < 0.001), but not in patients (*r*_*s*_= 0.13, *P* = 0.22). In serum, a similar pattern was found (presymptomatic carriers *r*_*s*_ = 0.46, *P* < 0.002, controls *r*_*s*_= 0.70, *P* < 0.001, patients *r*_*s*_= 0.23, *P* = 0.19). Females and males showed similar NfL levels (CSF *P* = 0.18, serum *P* = 0.08). CSF NfL levels in patients correlated positively with CDR and CDR‐SB, but not with MMSE or disease duration (Table 2, Fig. 2B); serum NfL correlated positively with CDR‐SB and not with MMSE or disease duration (Table 2). CSF NfL in four of the six patients with concomitant ALS fell in the highest 20% (Fig. 1A). Associations between NfL concentrations and estimated onset in presymptomatic carriers are displayed in Figure S2E and S2F.

**Table 2:** Association between neurofilament light chain levels (in cerebrospinal fluid [CSF] and serum) and clinical characteristics or scales in patients

	CSF			Serum		
	*r*_*s*_	*P*	*n*	*r*_*s*_	*P*	*n*
Disease duration	−0.07	0.50	84^a^	−0.33	0.06	34^a^
MMSE	−0.19	0.14	66	−0.28	0.13	30
CDR	0.33	0.04	40	0.36	0.08	25
CDR‐SB	0.60	0.001	27	0.53	0.02	19

MMSE, mini‐mental state examination; CDR, clinical dementia rating scale; CDR‐SB, clinical dementia rating scale sum of boxes.

a In two patients with CSF and one patient with serum, disease onset was unknown.

### CSF NfL levels versus brain volumes

Whole‐brain volume as a percentage of TIV was lower in patients than in presymptomatic carriers (*P* < 0.001) and lower in presymptomatic carriers than in controls (*P* = 0.04). Cortical volumes were lower in patients than in presymptomatic carriers in all investigated areas, except for occipital (all areas *P* < 0.001), without differences between controls and presymptomatic carriers.

CSF NfL in carriers negatively correlated with whole‐brain volume (Fig. 2C) and with frontal, temporal, parietal, insular and cingulate cortices (Table 3 and Fig. S4), indicating smaller volumes in case of higher CSF NfL. The analysis of patients only (*n* = 28) yielded significant negative correlations for whole brain, frontal cortex, and insular cortex. In presymptomatic carriers, negative correlations were found for whole brain and frontal, temporal, and parietal cortices. Subgroup analyses of scans from presymptomatic and symptomatic carriers combined and a CSF‐MRI interval of 90 days or less, as well as correction for gender and age showed similar patterns, albeit with lower correlation coefficients in the latter. Similar results were obtained after correction for study site. Unexpectedly, a positive correlation between NfL CSF and occipital cortex volume was found in the patient group.

**Table 3:** Correlations of cerebrospinal fluid (CSF) neurofilament light chain (NfL) with MRI volumes

		Cross‐sectional MRI			Longitudinal MRI
		All carriers, *n* = 55	Patients, *n* = 28	Presymptomatic carriers, *n* = 27	Annualized atrophy rate, all carriers, *n* = 17
Whole‐brain volume	*r*_*s*_	−0.78	−0.66	−0.43	0.79
	*P*	<0.001	<0.001	0.03	<0.001
Frontal	*r*_*s*_	−0.72	−0.54	−0.59	0.64
	*P*	<0.001	0.003	0.001	0.006
Temporal	*r*_*s*_	−0.51	−0.001	−0.50	0.74
	*P*	<0.001	1.00	0.008	0.001
Parietal	*r*_*s*_	−0.67	−0.24	−0.41	0.76
	*P*	<0.001	0.23	0.03	<0.001
Occipital	*r*_*s*_	0.004	0.56	−0.28	0.48
	*P*	0.98	0.002	0.16	0.05
Cingulate	*r*_*s*_	−0.43	−0.32	−0.21	0.72
	*P*	0.001	0.10	0.29	0.001
Insula	*r*_*s*_	−0.63	−0.59	−0.24	0.83
	*P*	<0.001	0.001	0.23	<0.001

Correlations of CSF NfL with whole‐brain and gray matter volumes at baseline are displayed in the first three columns. Correlations of CSF NfL with annualized atrophy rate from longitudinal scans are displayed in the last column.

In the subgroup of carriers with a follow‐up scan after CSF collection (7 patients and 10 presymptomatic carriers, median time between scans 1.1 years [interquartile range 1.0–2.1]) we found significant correlations between CSF NfL and annualized rate of atrophy for whole brain, frontal, temporal, parietal, cingulate, and insular cortices (Table 3 and Fig. 2D).

### Diagnostic performance

ROC analyses on CSF NfL levels showed a high AUC to separate patients both from controls (AUC 0.99 [95% CI: 0.97–1.00]) and from presymptomatic carriers (AUC 0.97 [0.94–0.99]), with a sensitivity of 84% and specificity of 100% for a cut‐off level of 2165 pg/mL (Fig. 1A). Lower AUCs, although not significantly lower, were found for serum NfL (patients versus controls 0.97 [0.93–1.00], patients versus presymptomatic carriers 0.93 [0.87–0.98]). Serum NfL had a sensitivity of 77% and a specificity of 98% to separate patients from presymptomatic carriers (cut‐off level of 18.0 pg/mL, Fig. 1C). To separate presymptomatic carriers from controls, CSF NfL levels showed an AUC of 0.65 (0.53–0.77) with a sensitivity of 40% and a specificity of 94% for a cut‐off level of 1066 pg/mL (Fig. S2A); the AUC of serum NfL for controls versus presymptomatic carriers was 0.63 (95% CI: 0.51–0.75, sensitivity 34% and specificity 97% at a cut‐off level of 8.3 pg/mL, Fig. S2C).

### Survival analyses

The median survival after CSF collection of deceased patients was 3.6 years (range 0.4–8.1, *n* = 34), the median follow‐up of alive patients was 2.8 years (range 0.4–10.7, *n* = 38). High CSF NfL levels were associated with a poor survival (estimated hazard ratio [HR] of 2.21 (95% CI: 1.30–3.77), *P* = 0.004, corrected for age and disease duration, Fig. 2E). This association was most prominent in *C9orf72* cases, even after correction for ALS (estimated HR 24.59, *P* = 0.02, corrected for ALS, age, and disease duration). Dividing the cohort into two groups gave similar results; dividing into four groups gave major overlap in CIs. Cox regressions on ‘raw’ CSF NfL confirmed the association with mortality: HR 1.02 for each increase in 1000 pg/mL (*P* < 0.001). Serum NfL was also associated with survival (estimated HR on tertiles 3.10, 95% CI: 1.09–8.76, *P* = 0.03, Fig. 2F, 14 deceased and 21 alive; estimated HR on ‘raw’ serum NfL 1.02, *P* = 0.02); gene‐specific analyses in serum yielded no significant results.

### Longitudinal samples

Longitudinal CSF samples of the two *GRN* converters, showed a three‐ to fourfold increase in NfL levels over conversion into the symptomatic stage (interval 3.1 and 2.0 years respectively; Fig. S5), with a 5.8‐fold increase (from 9.5 to 55.3 pg/mL) in serum samples available in one converter. A decrease (−48% in 1 year) in CSF NfL of the third relative to the second sample was seen in the symptomatic stage of one converter. Longitudinal CSF samples of one patient and two presymptomatic carriers showed a 0.8–1.5‐fold change (Fig. S5); the CVs of all described longitudinal samples were below 5%.

## Discussion

The present study on a large cohort of carriers of pathogenic *GRN, MAPT* or *C9orf72* mutations showed eight‐fold higher CSF NfL levels in patients than in presymptomatic carriers and controls. CSF NfL discriminated presymptomatic carriers from patients and might be useful to determine conversion. Serum NfL correlated highly with CSF NfL and showed a similar elevation in patients. Additionally, NfL levels in patients correlated with disease severity, brain atrophy, annualized brain atrophy rate, and survival. Hence, NfL in CSF or blood has the potential to serve as a biomarker for clinical disease onset and severity with a prognostic value.

The finding of elevated CSF and serum NfL levels in patients, with a good diagnostic performance to separate them from presymptomatic carriers, confirms the earlier findings in small series of presymptomatic carriers.^10^, ^15^ The strong correlation between CSF and serum NfL levels, alike in ALS,^15^, ^18^ suggests a promising role for serum NfL as a biomarker, as blood collections are more patient friendly than lumbar punctures. The trend for a lower correlation between serum and CSF NfL in controls than in mutation carriers is probably explained by the small group in combination with a suboptimal sensitivity of the serum assay in the lower range of values. Forthcoming new platforms have a higher sensitivity in this lower range; however, this will probably not influence the conclusions of this study, as genetic FTD patients showed high serum NfL levels.

The higher NfL levels in *GRN* patients than in *MAPT* patients are supported by earlier findings of higher CSF NfL in cases with TDP‐43‐pathology than with tau‐pathology,^34^ and suggest mutation‐specific underlying mechanisms. An intriguing question is whether NfL levels merely reflects the extent of neuronal cell death or white matter involvement, as has been reported in FTD‐*GRN*.^35^ Correlation of NfL levels with white matter damage has been found in FTD, AD, vascular dementia, and ALS.^10^, ^36^, ^37^ On the other hand, neurofilament proteins are also integral components of synapses with an important role in receptor‐specific synaptic plasticity.^17^ Therefore, mutation‐specific NfL elevation may reflect distinct pathophysiological mechanisms with a more white matter and/or synaptic origin of the disease process in *GRN* mutations. The wide range of NfL levels in our *C9orf72* patients correlated with the clinical phenotype, with mostly high levels in subjects with concomitant ALS and/or fast progression and low levels in patients with a slow progression. This is in line with high NfL levels in genetic ALS (half *C9orf72* carriers) and sporadic ALS, the latter correlating with a fast progression and DTI abnormalities.^15^, ^18^, ^37^ Although DTI analyses across multiple centers are at the moment challenging to harmonize, future DTI studies combined with NfL levels in the different genetic subtypes of FTD, may elucidate the relationship with white matter integrity.

The identified correlations of NfL levels with disease severity and survival in genetic FTD patients are also in line with earlier reports in sporadic FTD, AD, and ALS.^10^, ^11^, ^13^, ^18^ Specifically, the association of high NfL levels with a poor survival could serve as a meaningful prognostic clinical tool. The lack of correlation between NfL levels and age in patients as opposed to the controls and presymptomatic carriers, is likely explained by the magnitude of the disease effect outweighing the effect of age.

The negative correlation between CSF NfL and brain and cortical volumes is in line with findings in a cohort of mainly sporadic FTD patients in which a negative correlation with gray and white matter volume was found.^10^ This supports the hypothesis that NfL levels reflect the extent of neurodegeneration.^16^ So far, the positive correlation between CSF NfL and occipital volume in our patients is difficult to explain. Perhaps gene‐specific differences are underlying, since the occipital lobe is often affected in *C9orf72*,^38^ which is associated with relatively low NfL levels in current study, and spared in *GRN*, showing high NfL levels; however groups were too small for gene‐specific analyses. The correlation between CSF NfL and annualized rate of atrophy in the subset of carriers with two consecutive scans, supports the observed prognostic value of NfL levels in the cross‐sectional analysis. Larger future studies are needed to determine whether gene‐specific rates of atrophy, as found in the study by Whitwell et al.,^39^ could be correlated with corresponding NfL levels.

The observed three‐ to fourfold increase in CSF NfL levels within 3 years in our converters and normal levels over the entire presymptomatic phase in our large series of healthy mutation carriers, gives a first indication of the time period in which NfL increases. Although the time to onset is difficult to estimate, due to varying age at onset among families, we showed only a small increase in asymptomatic subjects approaching their estimated onset. The elevation in NfL levels suggests a rather explosive nature of the disease process, at least for *GRN* mutations, in which a rapid breakdown of the neuroaxonal compartment takes place, instead of a more linear disease progression. Similar dynamics are suggested in ALS.^40^ NfL levels in CSF and, according to our data, likely also in serum may thus serve as a biomarker for an active disease process coinciding with the onset of clinical symptoms in genetic FTD.

Major strengths of our study are the large series of presymptomatic carriers and patients with genetic FTD and the multimodal approach in correlating clinical and imaging data with a fluid biomarker. NfL determinations were performed in one laboratory which excludes an important source of variability.^41^ Additionally, studying genetic FTD allows us to investigate the earliest disease processes in subjects with a known underlying pathology, which is ideal to identify biomarkers. An important weakness in our study was the interval between collection of CSF, serum, and MRI scanning. However, results were similar in the carriers with an shorter interval between CSF sampling and MRI scanning as well as similar correlations in serum and CSF samples collected on the same day. Secondly, combining subjects from multiple centers resulted in variability regarding sample collection, however NfL measurements in CSF are known to be robust to preanalytical variables.^42^ Lastly, too few samples were available to draw conclusions on longitudinal dynamics and the meaning of the decrease in CSF NfL in one converter at a third time point. The relatively stable NfL levels over time in ALS might indicate that release and accumulation of NfL is counterbalanced by clearing mechanisms.^18^ Additionally, in multiple sclerosis CSF NfL have shown to dynamically decrease after therapeutical interventions, which suggests a potential to serve as a pharmacodynamic biomarker in FTD as well.^14^ Longitudinal NfL studies in CSF and serum in FTD are needed to determine (1) whether yearly NfL measurements are a robust biomarker for conversion; (2) changes throughout the disease process; and (3) the potential to measure pharmacodynamic response to interventions. In our opinion however, our cross‐sectional results clearly discriminated presymptomatic carriers from patients, making longitudinal studies interesting, but not necessary before the application in the clinic.

In conclusion, NfL in both CSF and serum is a promising biomarker for disease onset, severity, and survival in genetic FTD. Longitudinal studies are warranted to assess dynamics over time and thereby the usefulness of NfL for clinical trials in FTD.

## Author Contributions

LHM contributed to the study design, in the acquisition and interpretation of data, performed the statistical analysis and drafted the manuscript and figures. CB, JK, and CET contributed to the study design, in the acquisition of data (i.e. NfL determinations), and revised the manuscript. JCvS contributed to the study design and supervision and to the interpretation of data, and drafted the manuscript. All other authors (EGD, LCJ, RS‐V, CG, LB, RG, YAP, BB, DG, RL, MM, RV, IL, MO, JMP, RvM, SAR, MB, LO, VJ, DMC, SH, MJC, SO, KD, MNR, AP, ES, CF, MCT, FL, JDR) contributed to the study design, in the acquisition of data and in study coordination, and revised the manuscript.

## Conflicts of Interest

Dr. Cardoso reports grants from UK Medical Research Council (Centres of Excellence in Neurodegeneration grant), from Alzheimer's Research UK, Brain Research Trust, The Wolfson Foundation, during the conduct of the study. Dr. Cash reports grants from UK Medical Research Council (MR/M023664/1), Alzheimer's Research UK (ARUK‐PG2014‐1946), Brain Research Trust, The Wolfson Foundation, Anonymous Charity, during the conduct of the study. Dr. Teunissen reports personal fees from advisory board of Fujirebio and Roche, non‐financial support from research consumables from Euroimmun, IBL, Fujirebio, Invitrogen and Mesoscale Discovery, other from performed contract research for IBL, Shire, Boehringer, Roche and Probiodrug, outside the submitted work. Dr. Benussi reports grants from Ricerca Corrente, Italian Ministry, during the conduct of the study. Dr. Graff reports grants from Swedish Alzheimer foundation, Regional Agreement on Medical Training and Clinical Research (ALF) between Stockholm County Council and Karolinska Institutet, Strategic Research Program in Neuroscience at Karolinska Institutet, Karolinska Institutet's Doctoral Funding, Swedish Medical Research Council, Swedish Brain Power, Swedish Brain Foundation, Old Servants foundation, Gun and Bertil Stohne's foundation, King Gustaf V and Queen Victoria's Foundation of Freemasons, during the conduct of the study; grants from Hoffman La Roche Basel, outside the submitted work. Dr. Dopper reports grants from The Netherlands Organisation for Health Research and Development Memorabel grant 733050103, Netherlands Alzheimer Foundation Memorabel grant 733050103, Dioraphte Foundation grant 09‐02‐03‐00, Association for Frontemporal Dementias Research Grant 2009, The Netherlands Organization for Scientific Research grant HCMI 056‐13‐018, Netherlands Alzheimer Foundation, during the conduct of the study;. Dr. Ghidoni reports grants from Ricerca Corrente, Italian Ministry, during the conduct of the study;. Dr. LE BER reports grants from Program “Investissements d'avenir” ANR‐10‐IAIHU‐06, PHRC FTLD‐exomes, PHRC predict‐PGRN, ANR PrevDemAls, during the conduct of the study. Dr Kuhle's institution (University Hospital Basel) received in the last 3 years and used exclusively for research support: consulting fees from Novartis, Protagen AG; speaker fees from the Swiss MS Society, Biogen, Novartis, Roche, Genzyme; travel expenses from Merck Serono, Novartis; grants from ECTRIMS Research Fellowship Programme, University of Basel, Swiss MS Society, Swiss National Research Foundation, Bayer (Schweiz) AG, Genzyme, Novartis., outside the submitted work;. Dr. Papma reports grants from The Netherlands Organisation for Health Research and Development Memorabel grant 733050103, Netherlands Alzheimer Foundation Memorabel grant 733050103, during the conduct of the study;. Dr. van Swieten reports grants from The Netherlands Organisation for Health Research and Development Memorabel grant 733050103, Netherlands Alzheimer Foundation Memorabel grant 733050103, during the conduct of the study; grants from FORUM Pharmaceuticals, outside the submitted work;. Dr. Jiskoot reports grants from The Netherlands Organisation for Health Research and Development Memorabel grant 733050103, Netherlands Alzheimer Foundation Memorabel grant 733050103, during the conduct of the study;. Dr. Meeter reports grants from The Netherlands Organisation for Health Research and Development Memorabel grant 733050103, Netherlands Alzheimer Foundation Memorabel grant 733050103, Netherlands Alzheimer Foundation WE.09‐2014‐04, during the conduct of the study;. Dr. Otto reports grants from BMBF (Federal Ministry of Education and Research, Germany): Competence Net Neurodegenerative Dementias (project: FTLDc) and the JPND networks for standardization of biomarkers (BiomarkAPD, Sophia, PrefrontALS), during the conduct of the study;. Dr. Laforce reports grants from Novartis, outside the submitted work;. Dr. Rombouts reports grants from The Netherlands Organization for Scientific Research (VICI, grant number 016130677), during the conduct of the study;. Dr. Sánchez‐Valle reports grants from Fundacio La Marató de TV3, grants from National Institute of Health Carlos III (ISCIII) under the aegis of the EU Joint Programme ‐ Neurodegenerative Disease Research (JPND), during the conduct of the study;. Dr. Dick reports grants from UK Medical Research Council (Centres of Excellence in Neurodegeneration grant), Alzheimer's Research UK, Brain Research Trust, The Wolfson Foundation, during the conduct of the study;. Dr. Vandenberghe reports grants from Agency for Innovation by Science and Technology (IWT‐135043), during the conduct of the study;. Dr. Harding reports grants from UK Medical Research Council (Centres of Excellence in Neurodegeneration grant), Alzheimer's Research UK, Brain Research Trust, The Wolfson Foundation, during the conduct of the study;. Dr. Masellis reports grants from Canadian Institutes.

## Supporting information


**Data S1.** Methods.
**Figure S1.** Patient numbers per collected material and available MR‐imaging. Displayed numbers are after exclusion of outliers. *Three subjects were excluded from the analysis on the correlation between serum and CSF because the interval between serum and CSF collection was longer than 1 year (1 control, 2 presymptomatic carriers).
**Figure S2.** NfL levels in presymptomatic carriers and controls. NfL levels in (A) CSF and (C) serum by controls and presymptomatic carriers. Green dashed lines represent the cut‐off line to separate controls from presymptomatic carriers at 1066 pg/mL for CSF (sensitivity 40%, specificity 94%) and at 8.3 pg/mL for serum (sensitivity 34%, specificity 97%). NfL levels in (B) CSF and (D) serum in controls and presymptomatic carriers specified by genetic group (*GRN, C9orf72* and *MAPT*). Significances from the analysis of covariance analyses are displayed (corrected for age). Association between (E) CSF NfL and (F) serum NfL and time from estimated onset in controls (red circles) and presymptomatic carriers (*GRN* filled blue triangles, *C9orf72* filled blue squares, *MAPT* filled blue diamonds). One young individual is omitted from the graphs, but not from the analyses, to prevent disclosure of the genetic status. Presymptomatic carriers with CSF NfL values (*n* = 9) and serum NfL values (*n* = 14) of >2SD above the mean of controls were closer to or beyond the estimated onset (CSF mean 1.1 years and serum mean 0.8 years after estimated onset) than the presymptomatic carriers below that cut‐off (CSF mean 10.2 years and serum 9.1 years to estimated onset, both *P* < 0.001). In presymptomatic carriers, both CSF and serum NfL significantly correlated with time to onset or estimated onset (CSF *r*_*s*_= 0.69, *P* < 0.001 and serum *r*_*s*_= 0.57, *P* < 0.001). Ns, not significant.
**FigureS3.** Square root transformed NfL levels in presymptomatic carriers and patients. Square root of NfL in (A) CSF and (C) serum by controls, presymptomatic carriers and patients. Additionally, square root of NfL levels in (B) CSF and (D) serum specified by genetic group and clinical stage. Significances from the analysis of covariance analyses are displayed (corrected for age in all comparisons and additionally for disease duration in the comparisons between affected genes in patients). Ns, not significant; *P ≤ 0.05; **P ≤ 0.01; ***P ≤ 0.001.
**Figure S4.** Correlation between CSF NfL and MR‐imaging data. (A) Correlation of whole‐brain volume with CSF NfL in controls (red circles) and presymptomatic carriers (*GRN* blue filled triangles, *C9orf72* blue filled squares, *MAPT* blue filled diamonds). Correlations between CSF NfL and (B) frontal lobe volume and (C) temporal lobes volume in presymptomatic carriers (blue squares) and patients (orange triangles).
**Figure S5.** Longitudinal CSF NfL samples. Longitudinal samples of two converters (green and light blue lines), two presymptomatic carriers (dark blue lines) and one patient (orange line), plotted by time from onset or estimated onset in years.

## References

[1] Seelaar H , Rohrer JD , Pijnenburg YAL , et al. Clinical, genetic and pathological heterogeneity of frontotemporal dementia: a review. J Neurol Neurosurg Psychiatry 2011;82:476–486. 20971753 10.1136/jnnp.2010.212225

[2] Baker M , Mackenzie IR , Pickering‐Brown SM , et al. Mutations in progranulin cause tau‐negative frontotemporal dementia linked to chromosome 17. Nature 2006;442:916–919. 16862116 10.1038/nature05016

[3] Hutton M , Lendon CL , Rizzu P , et al. Association of missense and 5′‐splice‐site mutations in tau with the inherited dementia FTDP‐17. Nature 1998;393:702–705. 9641683 10.1038/31508

[4] DeJesus‐Hernandez M , Mackenzie IR , Boeve BF , et al. Expanded GGGGCC hexanucleotide repeat in noncoding region of C9ORF72 causes chromosome 9p‐linked FTD and ALS. Neuron 2011;72:245–256. 21944778 10.1016/j.neuron.2011.09.011 PMC3202986

[5] Renton AE , Majounie E , Waite A , et al. A hexanucleotide repeat expansion in C9ORF72 is the cause of chromosome 9p21‐linked ALS‐FTD. Neuron 2011;72:257–268. 21944779 10.1016/j.neuron.2011.09.010 PMC3200438

[6] Rohrer JD , Nicholas JM , Cash DM , et al. Presymptomatic cognitive and neuroanatomical changes in genetic frontotemporal dementia in the Genetic Frontotemporal dementia Initiative (GENFI) study: a cross‐sectional analysis. Lancet Neurol 2015;4422:1–10. 10.1016/S1474-4422(14)70324-2 PMC6742501 25662776

[7] Dopper EGP , Rombouts SA , Jiskoot LC , et al. Structural and functional brain connectivity in presymptomatic familial frontotemporal dementia. Neurology 2014;83:e19–e26. 25002573 10.1212/WNL.0000000000000583

[8] Bateman RJ , Xiong C , Benzinger TLS , et al. Clinical and biomarker changes in dominantly inherited Alzheimer's disease. N Engl J Med 2012;367:795–804. 22784036 10.1056/NEJMoa1202753 PMC3474597

[9] Tabrizi SJ , Scahill RI , Owen G , et al. Predictors of phenotypic progression and disease onset in premanifest and early‐stage Huntington's disease in the TRACK‐HD study: analysis of 36‐month observational data. Lancet Neurol 2013;12:637–649. 23664844 10.1016/S1474-4422(13)70088-7

[10] Scherling CS , Hall T , Berisha F , et al. Cerebrospinal fluid neurofilament concentration reflects disease severity in frontotemporal degeneration. Ann Neurol 2014;75:116–126. 24242746 10.1002/ana.24052 PMC4020786

[11] Skillback T , Farahmand B , Bartlett JW , et al. CSF neurofilament light differs in neurodegenerative diseases and predicts severity and survival. Neurology 2014;83:1945–1953. 25339208 10.1212/WNL.0000000000001015

[12] Steinacker P , Feneberg E , Weishaupt J , et al. Neurofilaments in the diagnosis of motoneuron diseases: a prospective study on 455 patients. J Neurol Neurosurg Psychiatry 2016;87:12–20. 26296871 10.1136/jnnp-2015-311387

[13] Zetterberg H , Skillbäck T , Mattsson N , et al. Association of cerebrospinal fluid neurofilament light concentration with alzheimer disease progression. JAMA Neurol 2016;73:60–67. 26524180 10.1001/jamaneurol.2015.3037 PMC5624219

[14] Kuhle J , Disanto G , Lorscheider J , et al. Fingolimod and CSF neurofilament light chain levels in relapsing‐remitting multiple sclerosis. Neurology 2015;84:1639–1643. 25809304 10.1212/WNL.0000000000001491 PMC4409586

[15] Weydt P , Oeckl P , Huss A , et al. Neurofilaments levels as biomarkers in asymptomatic and symptomatic familial ALS. Ann Neurol 2015;DOI:10.1002/ana.24552. 10.1002/ana.24552 26528863

[16] Petzold A . Neurofilament phosphoforms: surrogate markers for axonal injury, degeneration and loss. J Neurol Sci 2005;233:183–198. 15896809 10.1016/j.jns.2005.03.015

[17] Yuan A , Sershen H , Veeranna A , et al. Neurofilament subunits are integral components of synapses and modulate neurotransmission and behavior in vivo. Mol Psychiatry 2015;20:989–994. 10.1038/mp.2015.45 PMC4514553 25869803

[18] Lu C‐H , Macdonald‐Wallis C , Gray E , et al. Neurofilament light chain: a prognostic biomarker in amyotrophic lateral sclerosis. Neurology 2015;84:2247–2257. 25934855 10.1212/WNL.0000000000001642 PMC4456658

[19] Genetic FTD Initiative [Internet]. Available at: http://genfi.org.uk/ (accessed November 2015)

[20] Rascovsky K , Hodges JR , Knopman D , et al. Sensitivity of revised diagnostic criteria for the behavioural variant of frontotemporal dementia. Brain 2011;134:2456–2477. 21810890 10.1093/brain/awr179 PMC3170532

[21] Neary D , Snowden JS , Gustafson L , et al. Frontotemporal lobar degeneration: a consensus on clinical diagnostic criteria. Neurology 1998;51:1546–1554. 9855500 10.1212/wnl.51.6.1546

[22] Gorno‐Tempini ML , Hillis AE , Weintraub S , et al. Classification of primary progressive aphasia and its variants. Neurology 2011;76:1006–1014. 21325651 10.1212/WNL.0b013e31821103e6 PMC3059138

[23] Folstein MF , Folstein SE , McHugh PR . “Mini‐mental state”. A practical method for grading the cognitive state of patients for the clinician. J Psychiatr Res 1975;12:189–198. 1202204 10.1016/0022-3956(75)90026-6

[24] Morris JC . The clinical dementia rating (CDR): current version and scoring rules. Neurology 1993;43:2412–2414. 10.1212/wnl.43.11.2412-a 8232972

[25] Benussi L , Ghidoni R , Pegoiani E , et al. Progranulin Leu271LeufsX10 is one of the most common FTLD and CBS associated mutations worldwide. Neurobiol Dis 2009;33:379–385. 19101631 10.1016/j.nbd.2008.11.008

[26] Brooks BR , Miller RG , Swash M , Munsat TL .El Escorial revisited: revised criteria for the diagnosis of amyotrophic lateral sclerosis. Amyotroph Lateral Scler Other Motor Neuron Disord 2000;1:293–299. 11464847 10.1080/146608200300079536

[27] Modat M , Ridgway GR , Taylor ZA , et al. Fast free‐form deformation using graphics processing units. Comput Methods Programs Biomed 2010;98:278–284. 19818524 10.1016/j.cmpb.2009.09.002

[28] Cardoso MJ , Modat M , Wolz R , et al. Geodesic information flows: spatially‐variant graphs and their application to segmentation and fusion. IEEE Trans Med Imaging 2015;34:1976–1988. 25879909 10.1109/TMI.2015.2418298

[29] BrainCOLOR Protocol [Internet]. Available at: http://braincolor.mindboggle.info/docs/BrainCOLOR_cortical_parcellation_protocol.pdf (accessed November 2015).

[30] Neuromorphometrics [Internet]. Available at: http://www.neuromorphometrics.org:8080/seg/ (accessed November 2015).

[31] Gaiottino J , Norgren N , Dobson R , et al. Increased neurofilament light chain blood levels in neurodegenerative neurological diseases. PLoS One 2013;8:1–9. 10.1371/journal.pone.0075091 PMC3779219 24073237

[32] Limberg M , Disanto G , Barro C , Kuhle J . Neurofilament light chain determination from peripheral blood samples. Methods Mol Biol 2016;1304:93–98. 25687302 10.1007/7651_2015_206

[33] Youden WJ . Index for rating diagnostic tests. Cancer 1950;3:32–35. 15405679 10.1002/1097-0142(1950)3:1<32::aid-cncr2820030106>3.0.co;2-3

[34] Landqvist Waldö M , Frizell Santillo A , Passant U , et al. Cerebrospinal fluid neurofilament light chain protein levels in subtypes of frontotemporal dementia. BMC Neurol 2013;13:54. 23718879 10.1186/1471-2377-13-54 PMC3671150

[35] Caroppo P , Le Ber I , Camuzat A , et al. Extensive white matter involvement in patients with frontotemporal lobar degeneration. JAMA Neurol 2014;71:1562. 25317628 10.1001/jamaneurol.2014.1316

[36] Sjögren M , Blomberg M , Jonsson M , et al. Neurofilament protein in cerebrospinal fluid: a marker of white matter changes. J Neurosci Res 2001;66:510–516. 11746370 10.1002/jnr.1242

[37] Menke RAL , Gray E , Lu C‐H , et al. CSF neurofilament light chain reflects corticospinal tract degeneration in ALS. Ann Clin Transl Neurol 2015;2:748–755. 26273687 10.1002/acn3.212 PMC4531057

[38] Whitwell JL , Weigand SD , Boeve BF , et al. Neuroimaging signatures of frontotemporal dementia genetics: C9ORF72, tau, progranulin and sporadics. Brain 2012;135:794–806. 22366795 10.1093/brain/aws001 PMC3286334

[39] Whitwell JL , Boeve BF , Weigand SD , et al. Brain atrophy over time in genetic and sporadic frontotemporal dementia: a study of 198 serial magnetic resonance images. Eur J Neurol 2015;22:745–752. 25683866 10.1111/ene.12675 PMC4390434

[40] Benatar M , Wuu J . Presymptomatic studies in ALS: rationale, challenges, and approach. Neurology 2012;79:1732–1739. 23071166 10.1212/WNL.0b013e31826e9b1d PMC3468777

[41] Petzold A , Altintas A , Andreoni L , et al. Neurofilament ELISA validation. J Immunol Methods 2010;352:23–31. 19857497 10.1016/j.jim.2009.09.014

[42] Koel‐Simmelink MJA , Vennegoor A , Killestein J , et al. The impact of pre‐analytical variables on the stability of neurofilament proteins in CSF, determined by a novel validated SinglePlex Luminex assay and ELISA. J Immunol Methods 2014;402:43–49. 24275679 10.1016/j.jim.2013.11.008

