# Supplementary material for: Neurofilament light chain: a biomarker for genetic frontotemporal dementia
Source: Ann Clin Transl Neurol. 2016 Jul 1;3(8):623–36. doi: 10.1002/acn3.325 (PMC4999594; doi:10.1002/acn3.325)
Supplement: Supplementary file 1 — Data S1. Methods. Figure S1. Patient numbers per collected material and available MR‐imaging. Displayed numbers are after exclusion of outliers. *Three subjects were excluded from the analysis on the correlation between serum and CSF because the interval between serum and CSF collection was longer than 1 year (1 control, 2 presymptomatic carriers). Figure S2. NfL levels in presymptomatic carriers and controls. NfL levels in (A) CSF and (C) serum by controls and presymptomatic carriers. Green dashed lines represent the cut‐off line to separate controls from presymptomatic carriers at 1066 pg/mL for CSF (sensitivity 40%, specificity 94%) and at 8.3 pg/mL for serum (sensitivity 34%, specificity 97%). NfL levels in (B) CSF and (D) serum in controls and presymptomatic carriers specified by genetic group (GRN, C9orf72 and MAPT). Significances from the analysis of covariance analyses are displayed (corrected for age). Association between (E) CSF NfL and (F) serum NfL and time from estimated onset in controls (red circles) and presymptomatic carriers (GRN filled blue triangles, C9orf72 filled blue squares, MAPT filled blue diamonds). One young individual is omitted from the graphs, but not from the analyses, to prevent disclosure of the genetic status. Presymptomatic carriers with CSF NfL values (n = 9) and serum NfL values (n = 14) of >2SD above the mean of controls were closer to or beyond the estimated onset (CSF mean 1.1 years and serum mean 0.8 years after estimated onset) than the presymptomatic carriers below that cut‐off (CSF mean 10.2 years and serum 9.1 years to estimated onset, both P < 0.001). In presymptomatic carriers, both CSF and serum NfL significantly correlated with time to onset or estimated onset (CSF r s= 0.69, P < 0.001 and serum r s= 0.57, P < 0.001). Ns, not significant. FigureS3. Square root transformed NfL levels in presymptomatic carriers and patients. Square root of NfL in (A) CSF and (C) serum by controls, presymptomatic carriers and patients. [file ACN3-3-623-s001.pdf]

Supplementary File - Neurofilament light chain: a biomarker for genetic frontotemporal dementia

Supplementary Methods

Serum NfL concentrations were measured in duplicate by an earlier described, slightly modified, electrochemiluminescence (ECL) immunoassay with antibodies identical to those used in the CSF ELISA (Supplementary Methods).[1,2] The ECL assay was slightly modified: coating was done with 0.05 M carbonate-bicarbonate buffer (pH 9.6) at 4°C. Non-specific binding sites were blocked with 100 µl of TBS, containing 3% milk powder, per well for 1h. We used 25 µl of TBS containing 1% milk powder, 0.1% Tween 20 and 600/300 µg/ml HeteroBlock® (Omega Biologicals, Bozeman, MT, USA) as sample diluent. Calibrators were prepared in TBS containing 1% milk powder, 0.1% Tween 20 and 300 µg/ml HeteroBlock®. Samples below the lowest standard but above the signal of the blank were extrapolated from the standard curve, otherwise assigned a concentration of 0 pg/ml.[3] Intermediate precision/repeatability were 6.1%/3.7%, respectively (sample with mean concentration 72.8 pg/ml), 8.9%/7.1% (52.3 pg/ml) and 14.9%/9.8% (9.1 pg/ml) for the ECL assay.[4] All sample CVs of duplicate measurements were below 20.0% (median 4.7%).

Supplementary References

1. Gaiottino J, Norgren N, Dobson R, et al. Increased Neurofilament Light Chain Blood Levels in Neurodegenerative Neurological Diseases. PLoS One 2013;8(9):1–9.

2. Limberg M, Disanto G, Barro C, Kuhle J. Neurofilament Light Chain Determination from Peripheral Blood Samples. Methods Mol. Biol. 2016;1304:93–8.

3. Lee JW, Devanarayan V, Barrett YC, et al. Fit-for-Purpose Method Development and Validation for Successful Biomarker Measurement. Pharm. Res. 2006;23(2):312–328.

4. Andreasson U, Perret-Liaudet A, van Waalwijk van Doorn LJC, et al. A Practical Guide to Immunoassay Method Validation. Front. Neurol. 2015;6(August):179.

Supplementary Figures

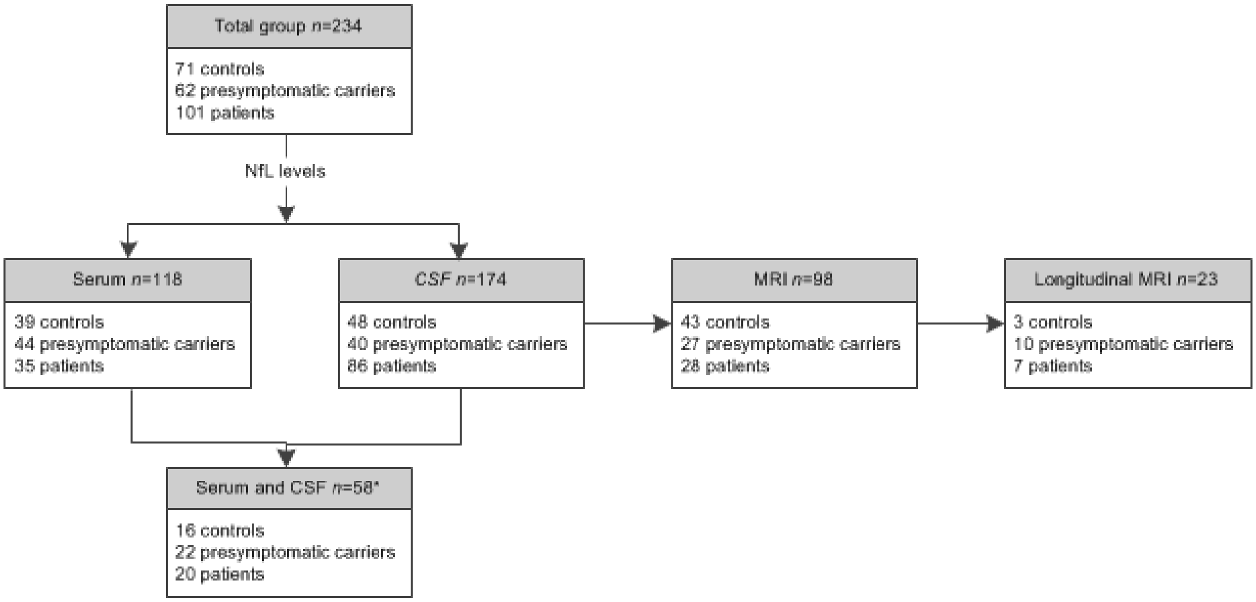

Supplementary Figure 1. Patient numbers per collected material and available MR-imaging. Displayed numbers are after exclusion of outliers. *Three subjects were excluded from the analysis on the correlation between serum and CSF because the interval between serum and CSF collection was longer than one year (1 control, 2 presymptomatic carriers).

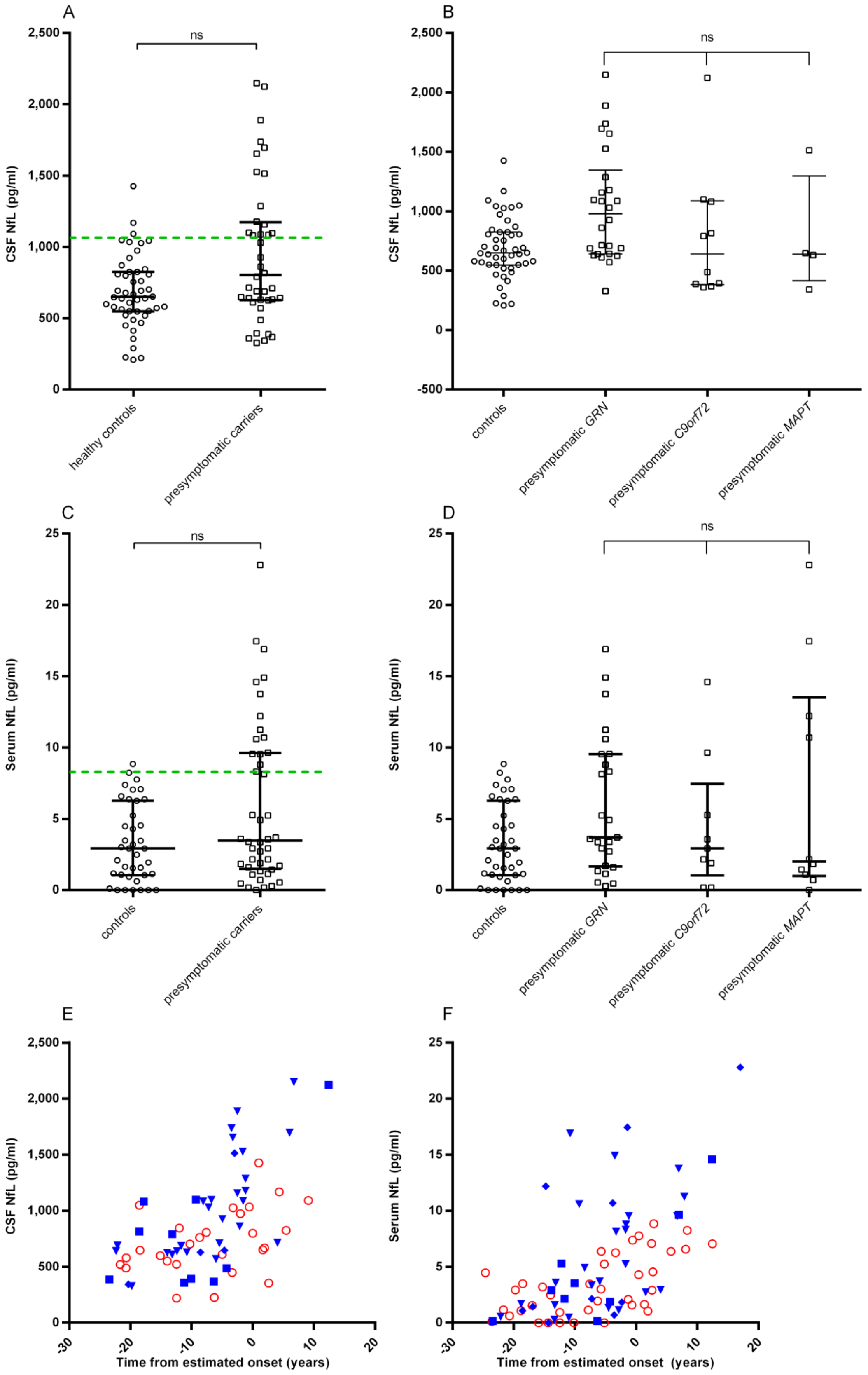
A
ns
CSF NfL (pg/ml)
healthy controls
presymptomatic carriers
B
ns
CSF NfL (pg/ml)
controls
presymptomatic GRN
presymptomatic C9orf72
presymptomatic MAPT
C
ns
Serum NfL (pg/ml)
controls
presymptomatic carriers
D
ns
Serum NfL (pg/ml)
controls
presymptomatic GRN
presymptomatic C9orf72
presymptomatic MAPT
E
CSF NfL (pg/ml)
Time from estimated onset (years)
F
Serum NfL (pg/ml)
Time from estimated onset (years)

Supplementary Figure 2. NfL levels in presymptomatic carriers and controls. NfL levels in (A) CSF and (C) serum by controls and presymptomatic carriers. Green dashed lines represent the cut-off line to separate controls from presymptomatic carriers at 1066 pg/ml for CSF (sensitivity 40%, specificity 94%) and at 8.3 pg/ml for serum (sensitivity 34%, specificity 97%). NfL levels in (B) CSF and (D) serum in controls and presymptomatic carriers specified by genetic group (*GRN, C9orf72* and *MAPT*). Significances from the ANCOVA analyses are displayed (corrected for age). Association between (E) CSF NfL and (F) serum NfL and time from estimated onset in controls (red circles) and presymptomatic carriers (*GRN* filled blue triangles, *C9orf72* filled blue squares, *MAPT* filled blue diamonds). One young individual is omitted from the graphs, but not from the analyses, to prevent disclosure of the genetic status. Presymptomatic carriers with CSF NfL values (n=9) and serum NfL values (n=14) of >2SD above the mean of controls were closer to or beyond the estimated onset (CSF mean 1,1 years and serum mean 0,8 years after estimated onset) than the presymptomatic carriers below that cut-off (CSF mean 10,2 years and serum 9,1 years to estimated onset, both p<0.001). In presymptomatic carriers, both CSF and serum NfL significantly correlated with time to onset or estimated onset (CSF r_s=0.69, p<0.001 and serum r_s=0.57, p<0.001). Ns, not significant.

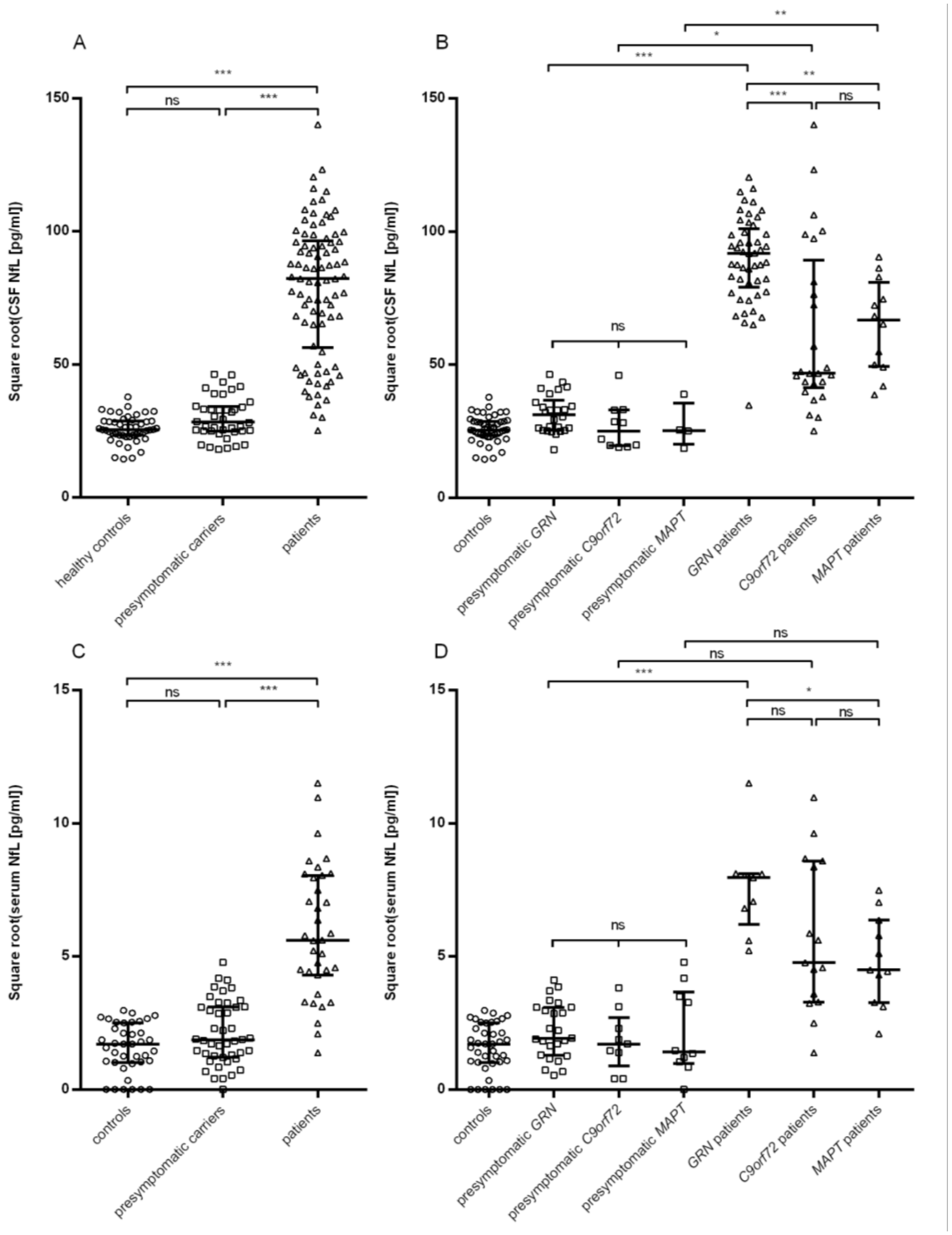

Supplemental Figure 3. Square root transformed NfL levels in presymptomatic carriers and patients. Square root of NfL in (A) CSF and (C) serum by controls, presymptomatic carriers and patients. Additionally, square root of NfL levels in (B) CSF and (D) serum specified by genetic group and clinical stage. Significances from the ANCOVA analyses are displayed (corrected for age in all comparisons and additionally for disease duration in the comparisons between affected genes in patients). Ns, not significant; *, $p \leq 0.05$; **, $p \leq 0.01$; ***, $p \leq 0.001$.

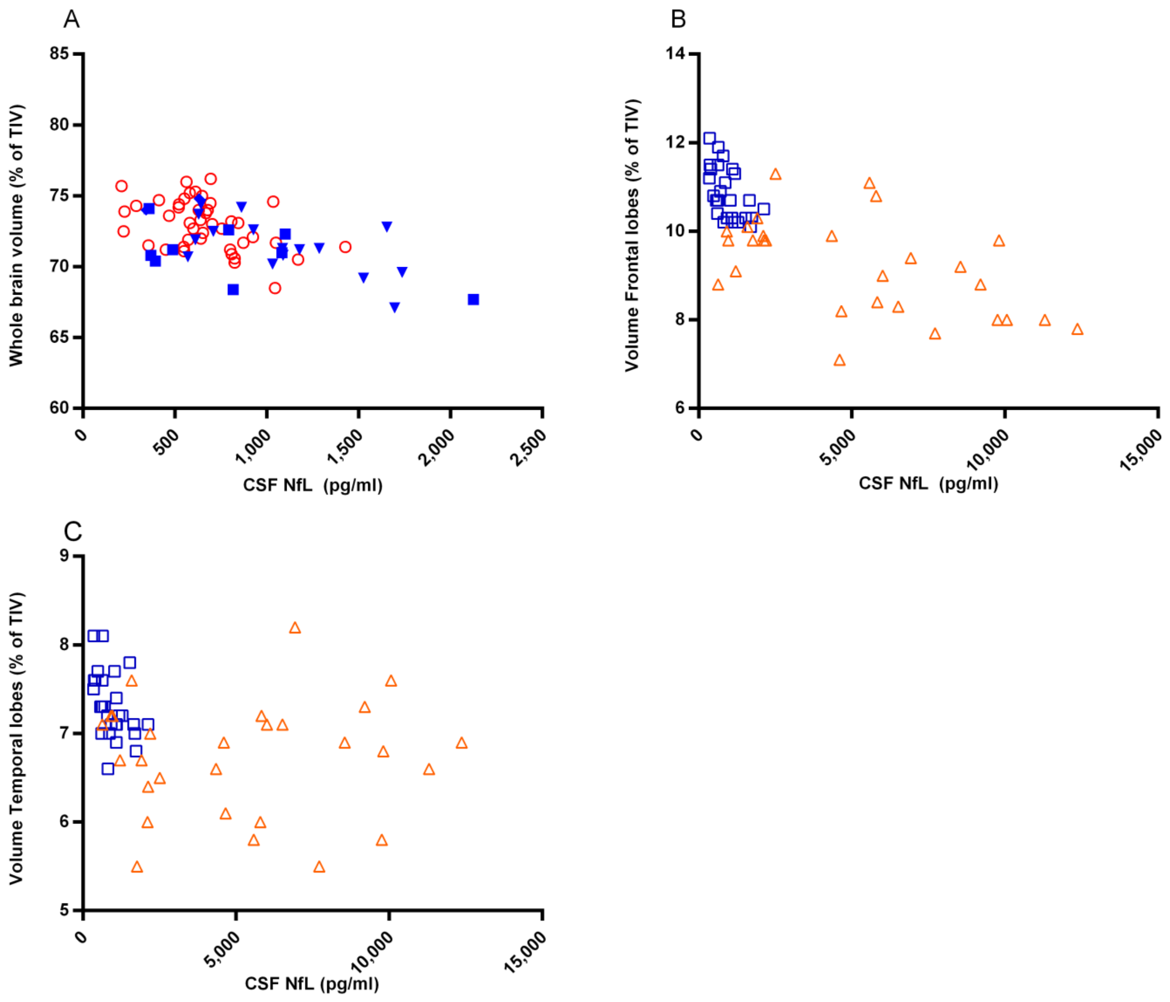

Supplementary Figure 4. Correlation between CSF NfL and MR-imaging data. (A) Correlation of whole brain volume with CSF NfL in controls (red circles) and presymptomatic carriers (*GRN* blue filled triangles, *C9orf72* blue filled squares, *MAPT* blue filled diamonds). Correlations between CSF NfL and (B) frontal lobe volume and (C) temporal lobes volume in presymptomatic carriers (blue squares) and patients (orange triangles).

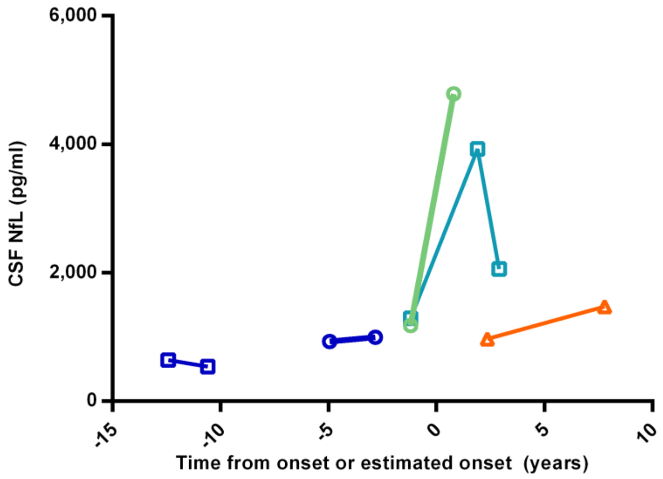

Supplementary Figure 5. Longitudinal CSF NfL samples. Longitudinal samples of two converters (green and light blue lines), two presymptomatic carriers (dark blue lines) and one patient (orange line), plotted by time from onset or estimated onset in years.
